# Editorial: The intersection of psychology, healthy behaviors, and their outcomes

**DOI:** 10.3389/fpsyt.2026.1809893

**Published:** 2026-02-25

**Authors:** Yijie Peng, Wai-kit Ming, Zheng Feei Ma, Yibo Wu

**Affiliations:** 1 Department of Nursing, the Fourth Affiliated Hospital of School of Medicine, and International School of Medicine, International Institutes of Medicine, Zhejiang University, Yiwu, China; 2 Guanghua School of Management, Peking University, Beijing, China; 3 Department of Infectious Diseases and Public Health, Jockey Club College of Veterinary Medicine and Life Sciences, City University of Hong Kong, Hong Kong, Hong Kong SAR, China; 4 Center for Public Health, School of Health and Social Wellbeing, University of the West of England, Bristol, United Kingdom

**Keywords:** digital health, health behaviors, health outcomes, mental health, psychological factors, public health, social determinants of health

## 1 Introduction

Health was once narrowly defined as the mere absence of disease (1, 2). Today, echoing the World Health Organization’s holistic vision, health is conceptualized as a dynamic interplay of physical, psychological, and social well-being (2, 3). Rather than viewing health as a static state of perfection, modern scholarship increasingly emphasizes the capacity for adaptation and resilience when facing biological, psychological, and social stressors (4–6).

This paradigm shift acknowledges that health outcomes, ranging from the management of chronic diseases to the severity of mental disorders and overall quality of life, emerge from complex interactions between individual traits, lifestyle choices, family dynamics, and broader socio-environmental contexts (7–10). Given the escalating global burden of mental and chronic conditions, elucidating the pathways toward these specific health endpoints has never been more imperative.

**Figure 1:**
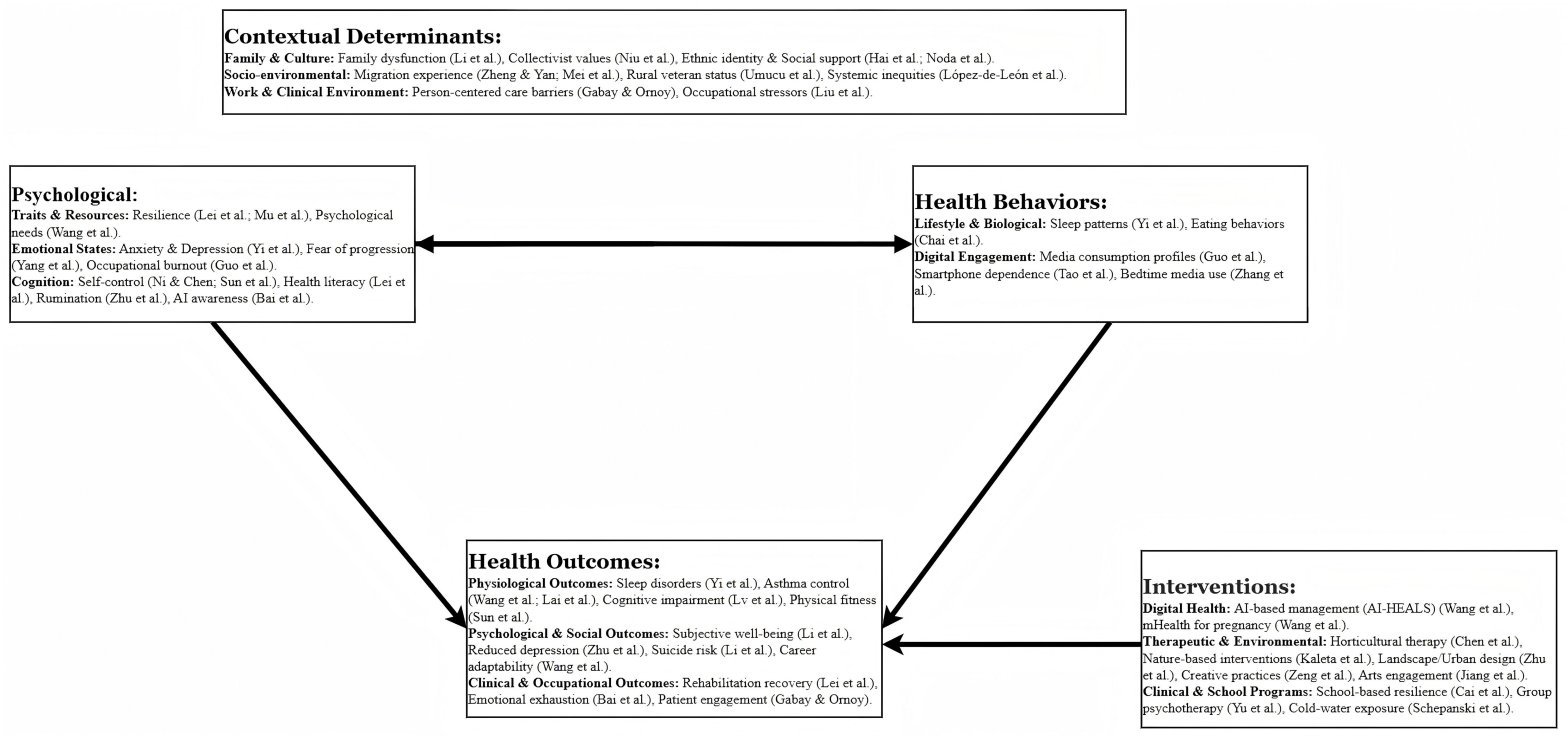
Overview of the intersection of psychology, healthy behaviors, and their outcomes.

This Research Topic, “The Intersection of Psychology, Healthy Behaviors, and Their Outcomes,” curates a substantial collection of 138 articles. It integrates a diverse body of evidence spanning the entire life course and encompassing populations from clinical patients to the general workforce. Collectively, these studies offer a comprehensive examination of how psychological states and daily behaviors intersect to shape tangible health outcomes, providing critical insights for future public health practice and policy (**Figure 1**).

## 2 From psychological states and behaviors to health outcomes

This Research Topic highlights how daily behaviors translate into measurable health outcomes, with physical activity serving as a pivotal determinant of psychological well-being via cognitive and emotional mechanisms. For instance, Zhu et al. identified rumination as a key mediator, showing that exercise negatively predicts depression. Similarly, Ni and Chen found that physical activity is associated with enhanced interpersonal competence through the mediating roles of self-control and emotional management. Meanwhile, Sun et al. demonstrated that specific high-intensity interval training combining online and offline sessions improves physical fitness and self-control in college students. Furthermore, Wang et al. reported that practices such as Tai Chi do not merely promote general activity but lead to the specific outcome of improved career adaptability alongside the satisfaction of basic psychological needs. Li et al. found that for junior high school students, participation in extracurricular sports is a direct predictor of subjective well-being, especially when combined with active engagement in physical education.

In addition to physical activity, sleep represents a critical behavioral domain where biological vulnerability and psychological stress converge to determine physiological outcomes. Yi et al. investigated this deep biological basis among mental workers, revealing a gene-environment interaction in which specific genotypes, such as PER2, when coupled with anxiety and depression, substantially increase the risk of sleep disorders. This interplay is further substantiated by evidence linking sleep disturbances to a range of systemic health consequences, including asthma exacerbation (Lai et al.) and impaired cognitive ability (Lv et al.)

In the digital context, several studies examine how media-related behaviors manifest as psychological outcomes. Moving beyond simple usage frequency, Guo et al. employed latent profile analysis on over 11,000 residents. They found that specific engagement profiles, such as “Omni-Media Users,” resulted in significantly higher levels of anxiety, suggesting that the mode of engagement is a critical determinant of psychological distress. This link to negative outcomes is echoed by Tao et al., who identified smartphone dependence as a contributor to negative emotions, and Zhang et al., who established an association between bedtime media exposure and fatigue. Conversely, psychological states act as potent antecedents to health-compromising behaviors that lead to poor physical outcomes. For example, Chai et al. revealed the relationship between stress, anxiety, and unhealthy eating behaviors, identifying that anxiety acts as a mediator through which high levels of stress lead to unhealthy eating behaviors.

## 3 Sociocultural determinants of health outcomes

Health outcomes are profoundly shaped by social, cultural, familial, and broader environmental contexts. Li et al. identified family dysfunction as a significant risk factor for the devastating outcome of suicide among children, uncovering a sequential pathway involving non-suicidal self-injury. Complementing this family-level perspective, Niu et al. introduced a cultural dimension, showing that undergraduates with stronger collectivist values are more susceptible to parental influence, which subsequently shapes their psychological adjustment. These findings highlight that sociocultural context is a determinant of adaptive outcomes, as further demonstrated by studies examining how ethnic identity, individualism, social support, and societal trust influence psychological adaptation and well-being (Hai et al., Liu et al., Noda et al.).

Extending beyond family and cultural contexts, broader socioeconomic conditions play a crucial role in determining population health outcomes. Research in this Research Topic highlights the unique challenges faced by diverse groups, including those in internal and international migration contexts (López-de-León et al., Mei et al., Zheng and Yan), individuals with disabilities (Umucu et al.), and veterans living in rural areas (Umucu et al.). These studies demonstrate how social displacement, housing status, and systemic inequities intersect with mental health to produce disparities in health outcomes.

## 4 Clinical and occupational outcomes: the role of adaptation

Psychological and behavioral factors intersect most clearly during adversity, as internal resources dictate clinical and occupational outcomes. In the clinical domain, Lei et al. and Mu et al. investigate how resilience and adaptive capacity shape recovery trajectories and psychological outcomes in patients with cardiac conditions. Focusing on stroke survivors, Lei et al. demonstrate that perceived risk of recurrence enhances health management awareness, with health literacy playing a mediating role in this process, which is a critical predictor of long-term rehabilitation outcomes.

Similarly, Gui et al. emphasize the buffering role of family support in alleviating death anxiety among breast cancer patients, while Zou et al. highlight that social isolation is a significant risk factor for depression in colorectal cancer patients. Across conditions such as hypertension, lymphoma, and other chronic diseases, a consistent pattern emerges where psychological factors such as Type D personality, interoceptive awareness, coping styles, and social support are closely associated with patient outcomes, including quality of life and disease management efficacy (Li et al., Yang et al., Yu et al., Zhu et al.). Furthermore, Gabay and Ornoy demonstrate that environmental factors, such as hospital-issued gowns, can influence patients’ sense of control and impact their engagement in care.

In occupational settings, the mental well-being of the workforce is a paramount health outcome in itself. Liu et al. reported a gradual deterioration in the mental health of Chinese nurses over a two-year longitudinal study, with psychological distress rising from 27.7% to 57.6%. Their findings identify higher stress coping scores as an independent risk factor for increased distress and PTSD. Their results suggest that maladaptive coping strategies were associated with higher distress levels. New technological stressors also impact occupational health outcomes, as Bai et al. revealed that “AI awareness” can deplete employees’ resources and trigger emotional exhaustion. In high-risk industrial settings, Guo et al. identified anxiety as a key mediator linking occupational burnout to sleep quality disturbances.

## 5 Interventions designed to improve health outcomes

Responding to these findings, this Research Topic emphasizes scalable interventions designed to positively influence health outcomes. Within digital health, Wang et al. introduced “AI-HEALS,” which is an artificial intelligence-based system aimed at improving asthma management, and proposed a protocol using mobile health technologies to support high-risk pregnant women, targeting improved prenatal outcomes.

Beyond digital approaches, several studies focus on therapeutic settings. Chen et al. conducted a meta-analysis confirming the efficacy of horticultural therapy in reducing depressive symptoms. Zhu et al. quantified visual landscape features to identify design thresholds associated with perceived security, linking urban design to psychological well-being. Additional studies document that nature-based interventions, school-based resilience programs, and creative practices are increasingly being explored for their potential to improve mental health outcomes (Cai et al., Jiang et al., Kaleta et al., Zeng et al.). Specific therapeutic modalities, such as group psychotherapy (Yu et al.) and cold-water exposure (Schepanski et al.), were also highlighted for their potential to enhance clinical outcomes.

Collectively, the articles in this Research Topic demonstrate that physical health, psychological well-being, and social environments are fundamentally intertwined in shaping final health outcomes. The breadth of research presented here reinforces the need to move beyond disease-centered models toward holistic approaches. Future research should continue to investigate these intersections through longitudinal designs to better understand the causal pathways leading to specific health outcomes. Looking ahead, we propose the establishment of a global interdisciplinary collaborative network to integrate resources across psychology, medicine, and public health, accelerating the translation of research findings into practices that tangibly improve global population health.
